# Plasma Levels of Osteopontin and Vascular Endothelial Growth Factor in Association with Clinical Features and Parameters of Tumor Burden in Patients with Multiple Myeloma

**DOI:** 10.1155/2014/513170

**Published:** 2014-06-04

**Authors:** Toni Valković, Emina Babarović, Ksenija Lučin, Sanja Štifter, Merica Aralica, Sanja Pećanić, Irena Seili-Bekafigo, Antica Duletić-Načinović, Damir Nemet, Nives Jonjić

**Affiliations:** ^1^Department of Hematology, Rijeka University Hospital Centre, Krešimirova 42, 51000 Rijeka, Croatia; ^2^Department of Pathology, School of Medicine, University of Rijeka, Braće Branchetta 20, 51000 Rijeka, Croatia; ^3^Department of Laboratory Medicine, Rijeka University Hospital Centre, Krešimirova 42, 51000 Rijeka, Croatia; ^4^Department of Cytology, Rijeka University Hospital Centre, Krešimirova 42, 51000 Rijeka, Croatia; ^5^Department of Hematology, Zagreb University Hospital Centre, Kišpatićeva 12, 10000 Zagreb, Croatia

## Abstract

The aim of this pilot study was to determine the plasma levels of osteopontin (OPN) and vascular endothelial growth factor (VEGF) and find possible association between them and main clinical features and parameters of tumor burden in patient with multiple myeloma (MM). Plasma levels of OPN and VEGF were determined in 44 newly diagnosed MM patients and 24 healthy persons by ELISA method. These values were compared with the presence of anemia, renal dysfunction, and bone lesions as myeloma related clinical manifestations and with serum beta-2 microglobulin and Durie-Salmon clinical stage as prognosticators related to tumor mass. The value of OPN was significantly higher in MM patients with evident bone lesions (*P* = 0.03) and there was also a positive correlation with serum beta-2 microglobulin (*r* = 0.366; *P* = 0.04). Furthermore, patients with lower Durie-Salmon stage had significantly lower OPN and VEGF levels (*P* = 0.05; *P* = 0.04, resp.). Our preliminary results found positive association between plasma level of OPN, tumor burden, and bone destruction. Further analysis should provide information about the possible use of OPN as useful clinical biomarker for monitoring bone disease and tumor mass, as well as a prognostic factor, or a possible target for pharmacological intervention.

## 1. Introduction

Multiple myeloma (MM) is a common haematological neoplasm with heterogeneous clinical manifestations, course of disease, response to treatment, and survival [1]. This unpredictable biological behaviour is a consequence of remarkably interesting, complex, and still unclear biological interactions between neoplastic plasma cells and other components of bone marrow microenvironment. Despite the great improvement in antitumor and supportive therapy, MM still remains an incurable disease. Vascular endothelial growth factor (VEGF) is considered one of the most potent angiogenic promoters in many solid tumours [2–6]. According to previous studies, it plays an angiogenic and tumorigenic role in the pathophysiology of MM [7–9], but the significance of its plasma level is still not well recognized. VEGF is produced by malignant plasma cells, as well as various inflammatory and stromal cells, acting through autocrine and/or paracrine crosstalk via their VEGFR-1 and VEGFR-2 receptors [7, 8, 10, 11].

Osteopontin (OPN) is a multifunctional, acid-rich, noncollagenous glycol-phosphoprotein expressed in bone, which interacts with integrin and CD44 receptors [12–14]. The binding of OPN to these surface receptors can elicit extensive changes in cell functions, such as enhanced mobility and adhesion, accelerated growth and division, prolongation of cell survival, and angiogenesis [15]. It is involved in a variety of physiological and pathological processes including inflammation, ischemia-reperfusion, bone resorption, atherosclerosis, and tumour progression [15]. In many types of human cancers the overexpression of OPN in tumour tissue or in blood has been associated with more advanced disease and recently it has been shown that OPN has the value as a clinical tumour progression marker [16]. It is well known that different stromal elements such as endothelial cells, macrophages, especially osteoclasts, lymphocytes, smooth muscle cells, and myeloma cells have potential to secrete OPN [17, 18]. There is a growing evidence for the role of OPN in the bone destruction and angiogenesis of MM [18–21].

However, until now, only a very few studies have partially explored associations between plasma levels of VEGF and OPN with some of the clinical features and parameters of tumor burden in myeloma patients [18–22]. In this pilot investigation we tried to detect possible association of plasma OPN and VEGF with myeloma related clinical manifestations such as anemia, renal dysfunction, and bone disease, as well as with serum beta-2 microglobulin and Durie-Salmon clinical stage which are prognosticators related to tumour burden.

## 2. Patients and Methods

### 2.1. Patients

We retrospectively analyzed 44 newly diagnosed, previously untreated myeloma patients (21 males, 23 females; median age of 69 years; age range 44–86 years) and 24 age-matched healthy individuals as a control group (12 males, 12 females; median age of 67 years; age range 35–83 years). Diagnoses were established at the Department of Hematology, Clinical Centre Rijeka, during the period from 2010 to 2012, according to the International Myeloma Working Group Criteria [23]. The control group consisted of healthy volunteers who were treated in the outpatient clinic hematology because of altered blood findings but none of them had any hematological disease. Patients with liver or renal impairment, current or previous other malignancies, infectious diseases, or incapability to consent were excluded from the control group. Blood samples were collected at the time of diagnosis from all MM patients, before the initiation of any antimyeloma treatment, including supportive treatment (e.g., bisphosphonate administration). All blood samples, collected from patients and controls, were aliquoted into separate vials, stored at −20°C, and assayed at the end of the study, in order to avoid interassay variability. Written informed consent was obtained from each patient and healthy volunteer prior to their inclusion in the study. The study was approved by the local ethics committee.

Patients were categorized according to Durie-Salmon clinical stage [24]. Because of the small number of cases in each Durie-Salmon stage group, we grouped clinical stages I and II together and compared them to stage III with the intent of separating patients with the largest tumor mass. The main characteristics of our patients are summarized in Table 1.

The obtained plasma cytokine levels were correlated with main clinical manifestations in MM: anemia (hemoglobin 20 g/L below the lower limit of normal, defined as 138 g/L for men and 119 g/L for women), renal dysfunction (serum creatinine level above the upper limit of normal, defined as 117 *μ*mol/L for men and 96 *μ*mol/L for women), and bone disease (the presence of any lytic lesion or severe osteopenia with compressive fractures on standard radiographs of the bones). The values of OPN and VEGF were also correlated with serum beta-2 microglobulin and with Durie-Salmon clinical stage (stages I and II combined versus stage III) as a measure of tumor mass.

### 2.2. Measurement of Cytokines

The concentrations of OPN and VEGF were determined in plasma samples by enzyme-linked immunoassay (ELISA, Quantikine RD Systems, Minneapolis, MN, USA) according to the manufacturer's instructions. In brief, these assays employ the quantitative sandwich immunoassay technique. A monoclonal antibody specific for protein was precoated onto microplates. Standards and samples were pipetted into the wells. After binding and washing, an enzyme-linked polyclonal antibody specific for each growth factor was added to each well. After a wash to remove any antibody-enzyme reagent, a substrate solution was added to the wells and color developed in proportion to the amount of each growth factor that was bound in the first step. The color development was stopped and optical density of each well was measured using the microplate reader set at 450 nm. The concentration of each specific growth factor in each plasma sample was calculated from standard curves and reported in pg/mL for VEGF and ng/mL for OPN.

### 2.3. Statistical Analysis

Statistical analyses were performed using MedCalc for Windows, version 12.2.1.0 (MedCalc Software, Ostend, Belgium). The distribution of data was tested for normality using the Kolmogorov-Smirnov test. The measures of central tendency for continuous data such as OPN and VEGF values were compared by Student's *t*-test or Mann-Whitney *U* test, depending on data distribution. The independent *t*-test and Mann-Whitney *U* test were used to assess whether continuous variables differed significantly between categories (patients with bone lesions versus patients without bone lesions, Durie-Salmon clinical stages I and II versus stage III, patients with anemia versus patients without anemia, etc.). Correlation between continuous variables was studied using Pearson correlation. Statistical differences with *P* < 0.05 were considered significant.

## 3. Results

**Figure 1:**
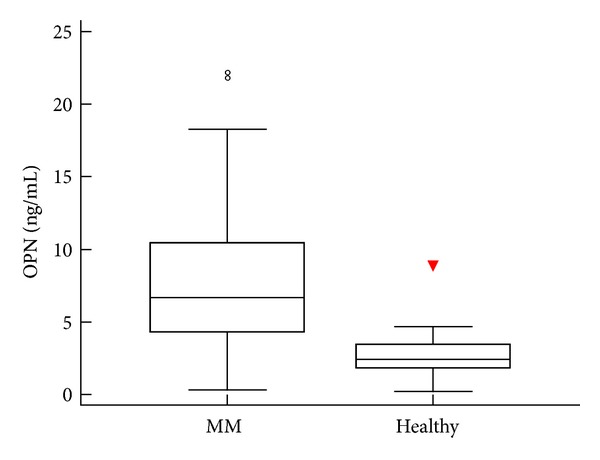
Comparison of plasma OPN levels between patients with MM and healthy volunteers who form the control group. The plasma concentrations of OPN were significantly higher in MM patients than in healthy volunteers (*P* < 0.0001, Mann-Whitney *U* test). The upper and lower borders of the box indicate the 75th and 25th percentiles, respectively, and the line in the box represents the median. The ends of the whiskers represent the minimum and maximum of all of the data excluding outliers. Outliers are plotted as individual points.

Certain amounts of both cytokines, OPN and VEGF, were detected in plasma samples from all patients. In addition, OPN value was significantly higher in MM patients (median 6.5 ng/mL, range 0.3–21.7 ng/mL) in comparison with the control group (median 2.4 ng/mL, range 0.2–8.9 ng/mL; *P* < 0.0001; Figure 1). Such differences were not observed regarding VEGF (median 52.5 pg/mL, range 5.3–178.9 pg/mL in MM patients versus median 60.5 pg/mL, range 12.2–205.6 pg/mL in control group; *P* = 0.67).

**Figure 2:**
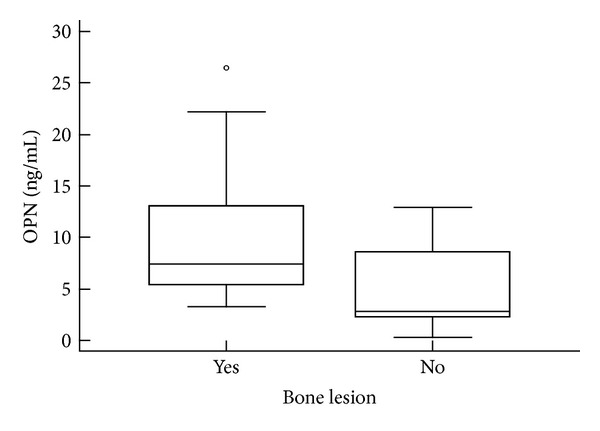
Comparison of plasma OPN levels between patients with bone disease and those without manifest bone lesions. The plasma concentrations of OPN were significantly higher in patients with bone disease (*P* = 0.03, Mann-Whitney *U* test). The upper and lower borders of the box indicate the 75th and 25th percentiles, respectively, and the line in the box represents the median. The ends of the whiskers represent the minimum and maximum of all of the data excluding outliers. Outliers are plotted as individual points.

In contrast with VEGF, plasma OPN levels were significantly higher in patients with evident bone lesions (*P* = 0.03; Figure 2). However, there were no statistically significant differences in levels of the analyzed cytokines in patients with anemia and renal insufficiency compared to those without these myeloma related complications. Further aim was to compare plasma levels of OPN and VEGF with parameters of tumor burden, and statistically significant differences among Durie-Salmon stages were observed. More specifically, patients in stages I and II had significantly lower plasma OPN (*P* = 0.05) and lower plasma VEGF (*P* = 0.04) values compared to patients in stage III of the disease. Furthermore, a significant positive correlation between plasma OPN concentration and serum beta-2 microglobulin level (*r* = 0.366; *P* = 0.04) was determined, while there was no correlation for VEGF plasma values. The results are summarized in Table 2.

## 4. Discussion

There is a certain body of evidence demonstrating the involvement of VEGF and OPN in angiogenesis and bone disease during MM progression. However, little is known about the possible clinical significance of plasma OPN and VEGF regarding other aspects of disease. As we know, myeloma is a collection of related disorders rather than a single disease. For that reason, the present study was conducted with the purpose of correlating serum concentrations of these cytokines with the most common presenting symptoms of MM (e.g., anemia, renal insufficiency, and bone disease), as well as with routine prognosticators beta-2 microglobulin and Durie-Salmon clinical stage, which reflect tumor burden.

The first result of this study revealed significantly higher plasma OPN in myeloma patients than in healthy controls. This finding agrees with some [25, 26], but not all, investigations [20, 27]. Furthermore, Saeki et al., Scudla et al., and Minarik et al. have observed differences in plasma OPN levels between monoclonal gammopathy of undetermined significance and MM [26, 28–30]. All of these results taken together implicate OPN in the biology of MM. Although several groups have demonstrated significantly increased plasma VEGF among myeloma patients in comparison with control groups [31–33], our current study and Sezer et al. [34] did not confirm this finding.

Our second finding revealed the associations of plasma concentrations of both VEGF and OPN with Durie-Salmon clinical stage. Plasma levels of both cytokines were significantly elevated in stage III compared with stages I and II. This positive association between cytokine values and Durie-Salmon clinical stage is not unexpected and is likely related to tumor burden. If that assumption is correct, the plasma levels of these cytokines (which can be produced by malignant plasma cells) might represent a measure of myeloma cell mass, not unlike the Durie-Salmon Staging System, which is based on a mathematical model for estimating the number of tumor cells. This result is in accordance with the finding that VEGF can act directly or indirectly as a growth factor for myeloma cells, which has been reported previously by some authors [7, 35, 36]. In addition, the present study demonstrated a significant positive association between OPN and another prognostic factor related to tumor burden and aggressive biological behavior of MM- beta-2 microglobulin, as previously described [20, 22]. However, we did not find the same association between VEGF and beta-2 microglobulin, which is intriguing and can be attributed to the relatively small number of cases included in this study. Still, it is possible that beta-2 microglobulin, in addition to myeloma cell mass and renal filtering capacity, might also reflect some other as-yet-unrecognized aspects of myeloma biology because, in contrast to Durie-Salmon clinical stage, it has retained prognostic value in the era of novel therapeutic agents.

We also observed significantly increased plasma OPN levels among myeloma patients with overt bone disease in comparison to those who had normal bone radiologic findings. Few groups have been able to demonstrate the same results [20, 22, 26]. Conversely, Robbiani and his group proposed that MM-derived OPN plays a critical role in bone disease by protecting bone from destruction [18]. Hence, the results of this study support the idea that OPN is an important pathophysiological factor in the biology of MM involved in bone disease. In addition, its positive association with parameters of tumor burden might suggest a role in the proliferation and survival of myeloma cells which should be explored in the future. All of this information affords us the opportunity to modulate its role in the tumor ecosystem through different pharmacological interventions.

According to our knowledge, only Dizdar et al. have investigated and proven a positive correlation between OPN and serum creatinine [22]. In contrast, we did not observe any relationship between plasma OPN level and renal impairment or anemia, which suggests that this multifunctional phosphoprotein is not relevantly involved in these aspects of MM. Moreover, our study reveals that plasma VEGF level was not associated with any of the main clinical features of MM or beta-2 microglobulin, a finding that is in concordance with the findings of other studies [32, 34]. Still, Di Raimondo et al. have observed that plasma VEGF levels correlate positively with beta-2 microglobulin, but not with osteolytic lesions, hemoglobin concentration, or creatinine concentration [9].

One question is whether plasma OPN (or VEGF) levels objectively reflect their concentrations in bone marrow, where the tumor is actually growing. Previous studies have shown that the concentrations of investigated angiogenic factors were higher in bone marrow but correlated significantly with plasma levels [9, 37].

Important limitations of present study are rather small sample size with missing clinical data for some patients, as well as its retrospective design, which limits any definite conclusions.

## 5. Conclusion

Our preliminary results support plasma OPN level as a possible marker of bone destruction. Although VEGF reportedly represents a critical angiogenic factor in the tumor microecosystem, the present study did not suggest that plasma VEGF level has similar clinical value to OPN. Furthermore, we did not observe associations between plasma levels of the investigated cytokines and other clinical features of MM, such as anemia or renal impairment. However, in the present pilot study, both cytokines were positively associated with tumor burden. Further prospective analysis of a larger group of patients should provide definitive information about the possible role of OPN as a useful clinical biomarker for monitoring bone disease and tumor mass, as well as a prognostic factor during the course of MM.

## Figures and Tables

**Table 1:** Clinical features of patients with multiple myeloma (MM) and healthy volunteers who form the control group.

Clinical features	Patients with MM (*N* = 44)	Healthy control group (*N* = 24)
Age and sex distribution	Cases	Cases
Male	21	12
Female	23	12
Age (years)	Median 69	Median 67
	Range 44–86	Range 35–83
Durie-Salmon stage	Cases	
I	7	
II	8	
III	29	
Renal dysfunction	Cases	
Yes	11	
No	32	
Anemia	Cases	
Yes	31	
No	13	
Beta-2 microglobulin	Cases	
Normal	9	
Increased	33	
Bone disease	Cases	
Yes	32	
No	10	

Note: renal dysfunction = serum creatinine level above the upper limit of normal; anemia = haemoglobin value 20 g/L below the lower limit of normal; beta-2 microglobulin = normal values less than 2.5 *μ*g/mL versus increased plasma values; and bone disease = presence of any lytic lesion or severe osteopenia with compressive fractures on standard radiographs of the bones.

**Table 2:** Comparison of measured plasma OPN and VEGF levels with clinical parameters in patients with MM.

	Durie-Salmon stage		*P* value	Bone lesions		*P* value	Anemia		*P* value	Renal dysfunction		*P* value
	I, II	III		Yes	No		Yes	No		Yes	No	
OPN (ng/mL)												
Median	5.65	8.9	**0.05**	7.4	2.8	**0.03**	7.6	6.6	0.29	12.6	7.05	0.13
Range	0.3–14.6	2.2–26.5		3.3–26.5	0.3–12.9		0.3–26.5	2.3–12.9		3.7–26.5	0.3–22.2	
VEGF (pg/mL)												
Median	35.9	60.15	**0.04**	48.1	55.75	0.79	57.4	47	0.43	46.9	59	0.51
Range	5.3–111.7	20.3–178.9		5.3–178.9	22.6–111.7		5.3–178.9	29.2–69.2		20.3–148.9	5.3–178.9	

## Ethnical Approval

Ethnical approval was received from the Ethnics Committees of the Rijeka University Hospital Centre and School of Medicine, University of Rijeka.
